# The craniofacial necrotizing fasciitis after a minor trauma in an elderly white woman

**DOI:** 10.4317/jced.51368

**Published:** 2014-07-01

**Authors:** Aleksandra Modlinska, Magdalena Osowicka, Tomasz Buss, Monika Lichodziejewska-Niemierko

**Affiliations:** 1 MD, PhD. Department of Palliative Medicine, Medical University of Gdansk, Poland

## Abstract

The term necrotizing fasciitis /NF/ was probably first described by Jones in 1871 as “hospital gangrene”. NF, with its fast spreading from the local infection to massive necrosis of the underlying tissues, ie. superficial fascia and subcutaneous layers, is a potentially fatal disease, unless diagnosed early and properly treated. NF is more frequent in frail patients with chronic debilitating illnesses, immune deficiencies or from a poor social background. Sixty percent of NF cases occur in females. Here we present a case of necrotizing fasciitis of the head and neck region after a minor trauma (phenol blocks due to severe neuropathic pain) in an 82-year-old female with the history of trigeminal neuralgia.

** Key words:**Necrotizing fasciitis, craniofacial infection, tissue necrosis.

## Introduction

The term necrotizing fasciitis [NF] was first used by Wilson in 1952, but as early as in 1871 Jones gave a description of what he termed “hospital gangrene” (1,2). NF is one of the most aggressive forms of soft tissue infections, involving the superficial fascia and subcutaneous layers (3,4,5). Diagnosis of NF is usually based on the clinical features, such as: fulminant progression, presence of grayish or black necrotic areas, easy separation of the superficial layer from the underlying tissues (5,6). NF spreads from a local infection site or is precipitated by a minor trauma, which provides a portal of entry for the infection. In every third patient the reason for NF cannot be pinpointed (3,5,6).

NF, if not diagnosed early and treated properly, is a potentially fatal disease (2,4,7,8), thus limited experience in NF diagnosis and treatment imposes a serious problem. The lower our limited experience in treatment of the disease, the more serious the problem may become (2,3). Here we present a case of necrotizing fasciitis of the head and neck region after a minor trauma in an elderly woman.

## Case Report

An 82-year-old woman, a resident of a social care institution, was hospitalized in the ophthalmology clinic, because of the total blindness of her left eye following central retinal artery thrombosis. The source of the embolism had not been established. One month later she was admitted to the internal medicine clinic because of asthenia, subfebrile condition, loss of appetite and difficulties in swallowing that had developed over several weeks. On admission the patient was weak, dehydrated with difficulties in verbal contact. Purpura was noted on the left side of the face.

Patient’s medical history was significant for trigeminal neuralgia. Phenol blocks, followed by diagnostic blocks with local anesthetic, had been used a few weeks before to relieve severe neuropathic pain.

Laboratory tests [compared with normal ranges in the elderly] showed the following abnormalities: leukocytosis was 18.5 K/mL with granulocyte predominance [74%]; CRP was 275 mg/L, Hb 11.4 g/dl [and 10.g/dL on the day of the discharge], serum sodium was low [133 mEq/L] accompanied by low potassium [3.25 mEq/L], there were also signs of an infection in the urinanalysis. Chest X-ray showed bilateral consolidations suggestive of pneumonia. Escherichia coli was cultured from urine and from blood. Subsequently amoxicillin with clavulanic acid, followed by cefotaxim, were prescribed, according to the antibiogram. No swab culture from the skin lesions was taken. Over the hospitalisation period a fast-advancing necrosis of the skin tissues of the face developed. The necrosis affected the left cheek with progression towards the temple, the left ala of the nose and the upper lip. A noma-like lesion was propounded. During the third week of the hospitalisation specimens of the tissue from the left cheek were collected. Histopathological evaluation revealed coagulative necrosis of the striped muscle and fat tissues with atypical cells among the purulent exudate - confirming the diagnosis of necrotizing fasciitis. Subsequently the patient was transferred to the palliative care institution.

On admission to the hospice the patient was in a moderately severe condition, emaciated, with effective breathing and circulatory functions and full logical contact but lowered mood. The necrosis of the cheek and the upper lip together with the fetid purulent discharge from the left corner of the mouth resulted in difficulties in the verbal contact and hampered feeding. The patient did not consent for the intra-oral examination due to severe pain.

A swab from the necrotic tissues was taken and ciprofloxacin and V-penicillin were administered in empirical treatment. E. coli was cultured and the antibiotics were continued for 2 weeks.

**Figure 1:**
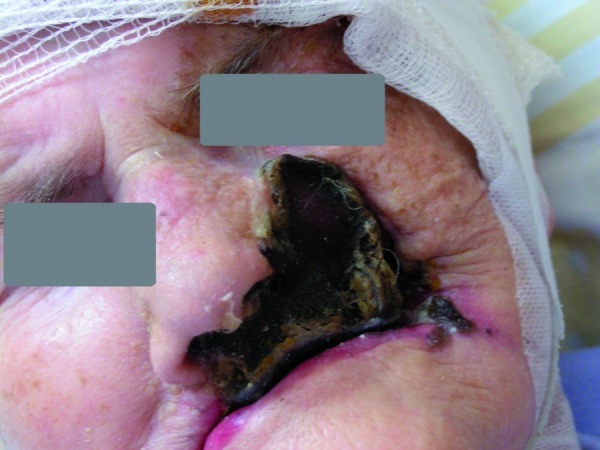
Expansion of the necrosis with spontaneous demarcation areas.

**Figure 2:**
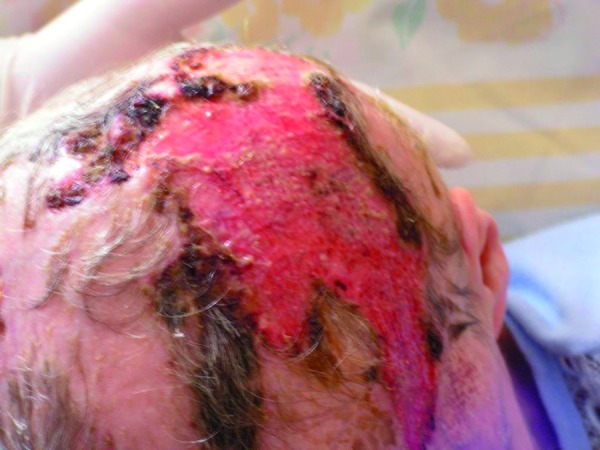
Numerous lesions on the hairy scalp.

**Figure 3:**
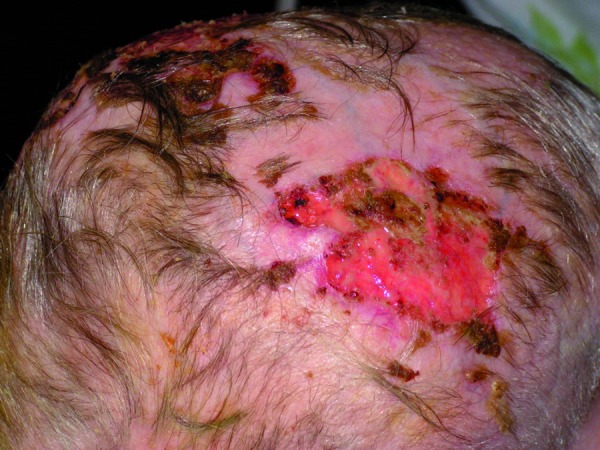
The hairy scalp over the necrotic region exfoliated from the galea.

The expansion of the necrosis was observed, with spontaneous demarcation of the necrotic areas of the upper lip, left corner of the mouth, left side of the nose and on the forehead (Fig. 1). Numerous new lesions were also noticed on the hairy scalp – initially resembling blood depositions, expanding into extensive necrotic lesions. Next the hairy scalp over the necrotic region spontaneously exfoliated from the galea (Fig. 2,3). There was a significant purulent discharge, from which Enterobacter cloacae, Klebsiella oxytoca, Proteus mirabilis and Candida albicans were cultured. Ciprofloxacin was administered again, along with metronidazole and fluconazole. Surgical demarcation was performed within the necrotic region.

Topical dressings with 10% povidone iodine and metronidazole were applied. Dressings were changed once a day, with a careful removal of the necrotic tissue. One miligrame of morphine sulphate was administered through an IV injection before each dressing change or wound debridement. Analgesic treatment included tramadol drops in doses increasing up to 100 mg every six hours, and carbamazepine. Corneregel [dexpanthenolum] and sulphacetamide were applied to the left eye and intravenous hydration with potassium supplementation was necessary to augment the semi-liquid diet.

CT scan of the scull and hyperbaric oxygen therapy were considered when the patient deteriorated and died on the 7th week from the admission.

## Discussion

NF most often affects the abdominal wall, the peritoneum and the lower extremities (3,4,7). The head and the neck are less common locations (5). NF in this region can present with two types: the cervical and the craniofacial [extremely rare] – with necrosis of the face, and sometimes of the eyelids and the scalp (3,4,5,7). Sixty percent of NF cases occur in females (7). NF is more frequent in patients with immune deficiencies and with chronic debilitating illnesses. NF onset is often preceded by a local infection. Usually, as it was in the case reported here, the disease affects patients coming from a neglected social background and extremely impoverished communities (3,4,7). Trauma has been reported to be an important factor in NF development.

Our patient developed craniofacial NF, most likely due to trauma elicited by the neurolytic injections administered for trigeminal neuralgia (9). The NF form discussed here is most commonly induced by repeated minor trauma followed by a local infection, e.g. skin, dental or sinus pathology and mucosal injuries (4,9). Local aesthetics neurolytic blocks of nerves are used to treat intractable neuropathic pain in the course of trigeminal neuralgia (10,11). These invasive procedures may cause significant complications i.e. orofascial infections and even corneal ulceration (9,12).

Estimated NF mortality is between 8.7% and 74% (7,13). Our patient was an old woman, from a neglected social background, suffering from anemia and pneumonia, the treatment was initiated after several weeks from the first symptoms – all these factors contributed to the initial risks (3,4,7). Chronic comorbidities, alcoholism, old age, female sex, a delay in referral to the specialistic centre /over 6 days/ are significant negative prognostic factors (5,12). Dehydration, bronchopneumonia due to aspiration, toxemia and complications of the disease are usually the main cause of death (12).

The significant portion of NF is caused by Enterobacteriaceae (4,7). Early detected ulceration should be treated with an intensive iv antibiotics with local disinfection, correct oral hygiene with adequate nourishment (3,4). Irrigation with physiological saline, hydrogen peroxide or boric acid solution should be performed and dressing should be changed using topical antiseptic agents, such as povidone iodine. The necrotized tissues should be delicately removed, in line with natural demarcation (7).

NF leaves serious damage, often requiring surgical reconstruction in survivors (3,13). Unfortunately, especially in advanced cases in the elderly, patients die despite aggressive treatment (3,12).
